# Media and information literacy among mothers in the 21st century: A scoping review protocol

**DOI:** 10.1371/journal.pone.0333890

**Published:** 2025-10-09

**Authors:** Yuan Jiang, Normah Mustaffa, Jamaluddin Aziz, XiaoChun Cai

**Affiliations:** 1 Center for Research in Media and Communication, Faculty of Social Sciences and Humanities, Universiti Kebangsaan Malaysia, Bangi, Selangor, Malaysia; 2 Center for Value Creation and Human Well-Being Studies, Faculty of Economics and Management, Universiti Kebangsaan Malaysia, Bangi, Selangor, Malaysia; Dow University of Health Sciences, PAKISTAN

## Abstract

**Background** Media and information literacy (MIL) is one of the most essential competencies people need in the 21st century. Although extensive research has focused on children and students, the MIL status of mothers, who are not only huge seekers and consumers of media and information but also crucial interventionists in family MIL, remains in its infancy.

**Objective** The objective of this scoping review is to systematically map the existing studies on mothers’ MIL in the 21st century. Specifically, it aims to perform a bibliometric analysis of relevant research; clarify the condition of mothers’ MIL in the 21st century; summarize the theoretical frameworks and measurement methodologies; and identify key influencing variables.

**Inclusion criteria** All primary or peer-reviewed articles on mothers’ MIL published after 2000 will be included, regardless of language or region. There are no participant restrictions, such as age, region, marital status, etc. All relevant concepts, such as “computer literacy,” “digital literacy,” “Information and Communications Technology (ICT) literacy,” “information literacy,” “MIL,” “media literacy,” “news literacy,” “social media literacy,” “technology literacy,” and “visual literacy” are included.

**Methods** The review will adhere to the JBI’s Scoping Review Guidelines and utilize the PRISMA-ScR reporting standards. A comprehensive search strategy based on the PCC framework will be applied across four databases: Web of Science, Scopus, EBSCOhost and Taylor& Francis. Two reviewers will separately search the databases in a two-round screening and extract data using a designated extraction instrument to respond to review queries. The search strategy and the instrument had been refined through a pilot test. A PRISMA flow diagram will be used to depict the entire screening procedure.

**Registration** This scoping review protocol has been registered in the Open Science Framework (https://doi.org/10.17605/OSF.IO/2N5V7).

## Introduction

Media and Information Literacy (MIL) is widely recognized as one of the must-have competencies that people need to possess in the 21st century [1,2]. It equips information consumers with the ability to identify credible information from misinformation and disinformation, make informed decisions, and develop critical thinking skills to address the growing challenges of the “information epidemic” [3–5]. Consequently, as a core component of students’ education and everyone’s lifelong learning [1], MIL has been extensively studied for decades, particularly among children, the elderly, students, teachers, and media or information professionals.

However, mothers—who are increasingly reliant on digital technology in the 21st century [6]—have received limited attention. Assessments of digital competency are also predominantly aimed at students or professionals rather than mothers [6]. But much data shows that the group of mothers cannot be ignored. In 2023, 90% of US mothers accessed the Internet using smartphones, spending an average of four hours and fifteen minutes a day [7]. They use the Internet to access educational resources, health information, and social support.

Additionally, mothers, as primary caregivers and media gatekeepers, are often considered to play a more important role in media intervention and MIL education for their children than fathers [14–16]. A growing number of research studies suggest that mothers’ MIL as an independent variable or a component of parental mediation [8], significantly influences their children’s MIL condition or media-related behaviors. These studies have concluded that mothers’ MIL can influence children’s dietary habits, social media use, digital media exposure, media dependence, mental development, and executive function, and risk behaviors such as e-cigarette consumption [9–14]. But evidence also shows that the mothers’ MIL level is not necessarily higher than their children’s [17], and inadequate MIL may have a detrimental rather than positive impact on their children [18]. Therefore, there is an urgent need to understand the level of mothers’ MIL and to look into how to improve it.

Although limited in number, some studies focus on how mothers’ MIL affects their own well-being. They have concluded that improving mothers’ digital literacy is crucial in reducing the negative impact of their social media use [19]; the health information literacy of mothers is related to their motivation to find maternal health information on the Internet [20]; enhancing media literacy among postpartum women is important for minimizing the negative effects of media use [21]; mothers’ improved access to information can help mitigate the negative effects of the Internet [2]; improving mothers’ media literacy could help to alleviate their parenting anxiety [22]; a portion of rural Chinese mothers use the media to maintain family and social relationships, seek self-identity and emotional adjustment, and also use the new media to obtain economic income [23]. In this way, we can affirm that because mothers’ MIL has impacts on themselves, their children, and families, we need to pay more attention to research on mothers’ MIL.

Despite the existing findings, research into mothers’ MIL is still in its infancy. Firstly, the number of relevant studies is modest, and the research field is fragmented. Secondly, there is no consensus on conceptual definitions or assessment frameworks among researchers. A comprehensive scoping review is thus necessary to map the knowledge base, highlight gaps, and support subsequent studies.

In order to prevent duplication, initial searches of Open Science Framework, Figshare, JBI Evidence Synthesis, Google Scholar, and Scopus have been conducted and have found no systematic or scoping reviews on mothers’ MIL. Thus, following the guidelines of JBI scoping reviews, this scoping review will examine mothers’ MIL in the information age of the 21st century. All relevant studies published in the 21st century, utilizing either qualitative or quantitative methodology, will be included without language restrictions. As the review will focus on empirical evidence, gray literature will be excluded this time.

## Review objectives

This scoping review aims to (1) conduct a bibliometric analysis of the relevant research; (2) clarify the definition and condition of mothers’ MIL; (3) identify the theoretical frameworks and methodological approaches used in these studies; and (4) determine key factors influencing mothers’ MIL. By mapping the research landscape in this field and uncovering the research gap, the review will propose recommendations for future research.

## Review questions

1. How many studies on mothers’ MIL have been published, and how have research trends evolved over time and across disciplines?

2. How is scholarly output on this topic distributed by country, institution, journal, and academic field?

3. Who are the most influential authors in this area of research?

4. Which articles and topics have had the greatest impact in the field of mothers’ MIL?

5. What theoretical frameworks and methodological approaches have been used to conceptualize and assess mothers’ MIL?

6. What are the reported conditions and influencing factors of mothers’ MIL in the 21st century?

## Inclusion criteria

Participants, Concepts, Context (PCC) was used as the framework for developing inclusion criteria for this scoping review [24].

### Participants

This review will include studies involving mothers of any age, marital status, or geographic background, encompassing both biological and adoptive mothers. Pregnant women who are explicitly identified as mothers, as well as postpartum women, will also be included without restriction.

### Concept

The concept of MIL is understood as an umbrella term encompassing various related literacies [25]. Accordingly, this review will include studies addressing “media literacy,” “information literacy,” “MIL,” “news literacy,” “digital literacy,” “ICT literacy,” “social media literacy,” “technology literacy,” “visual literacy,” and “computer literacy”.

### Context

Studies conducted in 21st-century information societies will be reviewed in this review. That means only literature published from the year 2000 onward will be considered.

### Types of sources

This scoping review will include all relevant primary, peer-reviewed studies, regardless of study design or methodology. Gray literature (e.g., policy reports, dissertations, conference proceedings) will be excluded to maintain a clear focus on peer-reviewed empirical evidence. This decision was made to ensure the quality, consistency, and traceability of the included evidence. While gray literature may offer additional insights, the inclusion of only peer-reviewed sources supports the scientific rigor, reliability, and replicability of the review findings, aligning with the objectives of mapping the empirical knowledge base in this field.

## Method

The scoping review will adhere to the Joanna Briggs Institute (JBI) scoping review methodology [24] and follow the PRISMA-ScR reporting guidelines [26]. Methodological rigor and transparency are ensured by using these frameworks. Two researchers and two experts in MIL will collaborate to resolve any disagreements during the selection and analysis processes. This protocol has been checked against the PRISMA-P checklist (see S2 Appendix) [27] and has been deposited in the Open Science Framework (https://doi.org/10.17605/OSF.IO/2N5V7).

### Search strategy

The search strategy will be executed in two phases. Initially, preliminary searches have been conducted in Google Scholar and Scopus databases to identify key terms and indexing practices. Based on this, and in consultation with two MIL experts, a comprehensive search strategy was formulated. A sample search string for the Web of Science database is presented in Table 1. A detailed search strategy for each database (Web of Science, Scopus, EBSCOhost and Taylor & Francis) is provided to ensure reproducibility (see S1 Appendix). In the second phase, two reviewers will search and screen reference lists for all included studies to identify additional eligible sources. This iterative process will continue until no new relevant records are found. There are no language restrictions during the search process.

**Table 1:** Web of Science Core Collection <2004 to April 28, 2025>.

Search	Query	Records retrieved
#1	TS=(“media and information literac*”) OR TS=(“media literac*”) OR TS=(“information literac*”) OR TS=(“digital literac*”) OR TS=(“news literac*”) OR TS=(“social media literac*”) OR TS=(“computer literac*”) OR TS=(“technological literac*”) OR TS=(“visual literac*”) OR TS=(“ICT literac*”) OR (“information and communication technology literac*”)	24390
#2	TS=(mother$) OR TS=(“female parent$”) OR TS=(mommy) OR TS=(mummy) OR TS=(mom) OR TS=(mum)	559731
#3	#1 AND #2	147

### Evidence source selection

Following the above two stages of screening, all evidence meeting the inclusion criteria will be imported to EndNote v.21 (Clarivate Analytics, PA, USA) for deduplication. After removing duplicates, detailed data of all evidence requiring further confirmation of relevance will be imported into JBI SUMARI (JBI, Adelaide, Australia) for subsequent review by the reviewers [28]. An initial pilot test will be conducted using the first 10 articles, during which two reviewers will independently evaluate and screen the titles, abstracts, and keywords adhering to the predefined inclusion criteria. They will subsequently check and adjust their screening results to ensure consistency in applying the criteria. In the formal review stage, the two independent reviewers will screen the full content of all evidence that pass the initial screening. Studies deemed ineligible will be excluded with documented reasons, which will be reported in the final report. Any disagreements during the screening process will be discussed by the two reviewers to resolve. If an agreement cannot be reached, a third expert specializing in media and information literacy will be consulted for adjudication. Additionally, as the first and fourth authors are native Chinese speakers with sufficient proficiency in English, and the second and third authors are native Malay speakers with English as their second language, articles in English, Chinese, and Malay can be reviewed directly. For literature published in other languages, professional translation services or consultation with multilingual researchers will be employed to ensure accurate interpretation and minimize language bias. Finally, a PRISMA flow diagram will be created and presented in the final scoping review [29].

### Data extraction

Two reviewers will separately extract data using the draft data extraction instrument in Table 2. This instrument was adapted from the JBI results extraction instrument template [24], tailored to the specific objectives of this scoping review, and validated through a pilot test.

**Table 2:** Draft data extraction instrument.

Scoping Review Details	
Scoping Review title	
Review objective	
Review question	
Inclusion/Exclusion Criteria	
Population	
Concept	
Context	
Types of evidence source	
Evidence source Details and Characteristics	
Citation details (e.g. author/s, date, title, journal, volume, issue, pages, and citation count)	
Primary affiliation of primary authors	
Subject area	
Country of origin	
Results extracted from source of evidence	
Research theme	
Research objectives	
Conceptual framework (including research variables)	
Theoretical framework	
Methodology	
Participants (e.g. age, number, identity)	
Results and key findings (including MIL levels, factors, strategies, impacts)	
Research limitations	

The extracted data will cover four main components:

(1) Scoping review details: title, objectives, and the review questions.

(2) Inclusion and exclusion criteria: descriptions of the population, concepts, context, and evidence sources types.

(3) Evidence source details and characteristics: citation details (e.g., authors, publication date, title, journal, volume, issue, page numbers, and citation count), the primary affiliation of the lead authors, subject area, and the country of origin.

(4) Extracted results, including the research themes, research objectives, conceptual frameworks (including research variables), theoretical framework, research methodologies, participant details (e.g., age, number, identity), key findings, and reported research limitations.

Among them, the key findings of the review can be categorized into four major areas: (1) mothers’ MIL level; (2) factors influencing maternal MIL, including demographic factors (e.g., maternal age, child age, education level, household income, and occupation) and sociocultural factors (e.g., national differences, urban–rural disparities, and regional variations in technological access and sociocultural settings); (3) strategies and interventions aimed at enhancing maternal MIL; (4) the roles and impacts of maternal MIL on individual and family outcomes.

If necessary, the draft data extraction instrument will be refined in the subsequent data extraction process. The revised version will be presented in the final report. Additionally, if there is a disagreement between the two reviewers after any session, they will reach an agreement through discussion and negotiation. If no agreement can be reached, third MIL experts’ advice will be heard.

### Data analysis and presentation

The extracted data will be started with a basic descriptive analysis presenting the results of the bibliometric analysis according to the JBI scoping review framework [30]. And the results of the bibliometric analysis will be presented in the form of tables or graphs to address and illustrate the relevant research questions. Additionally, evidence from all included studies will be summarized in a comprehensive table, supplemented by a narrative synthesis highlighting key findings and providing a critical discussion of the scoping review.

To address heterogeneity among the included studies, thematic coding and subgroup analysis will be conducted. Variations arising from differences in study populations, methodologies, cultural backgrounds, and contextual settings will be systematically managed by grouping the studies according to research themes, geographic region, cultural context, and participant demographics (e.g., children’s age). This structured approach will facilitate meaningful comparisons across diverse research contexts.

A combined approach incorporating thematic synthesis and a framework-based matrix will be adopted. Findings from both qualitative and quantitative studies will be coded and synthesized according to different research themes, which correspond to a predefined conceptual framework, including: (1) maternal MIL level, (2) influencing factors, (3) strategies and interventions, and (4) impacts and outcomes of MIL.This approach enables the integration of diverse forms of evidence without separating by study design, while ensuring that all results are meaningfully organized and compared within each thematic category. It also facilitates the identification of cross-cutting insights and research gaps, supporting a comprehensive and structured understanding of mother’s MIL.

### Study status and timeline

Data collection has been ongoing since April 2025 and will continue until the time of manuscript submission, with completion anticipated by May 2025. Data analysis is scheduled to begin in June 2025, and preliminary findings are expected by August 2025. At the time of submission, no interim or final results have been produced.

### Limitation

This scoping review protocol acknowledges several anticipated limitations that may influence the comprehensiveness and interpretability of the findings.

**Scope of literature inclusion.** Despite employing a comprehensive, multi-database search strategy, the exclusion of gray literature may lead to the omission of potentially relevant insights from non-peer-reviewed sources. Although this decision was made to prioritize methodological consistency and focus on empirical evidence with higher levels of academic validation, future research may build upon this work by broadening the inclusion criteria to incorporate diverse sources and capture a more holistic view of the research landscape.

**Potential risk of language bias.** This review aims to include studies in all languages, with support from multilingual researchers and professional translation services. However, for literature published in languages other than English, Chinese, and Malay, practical limitations in translation resources may still lead to language bias. While every effort will be made to minimize this risk, the possibility of misinterpretation cannot be entirely ruled out.

**Conceptual and measurement heterogeneity of MIL.** The conceptualization and measurement of Media and Information Literacy (MIL) vary considerably across studies, with no universal standard. Such heterogeneity may complicate the comparison and synthesis of findings. Although thematic analysis and subgrouping will be used to manage variability, inconsistent terminology and assessment tools may still affect the generalizability of results.

**Time restriction.** This review includes only studies published from the year 2000 onward to focus on MIL in the 21st-century information society. While this approach ensures relevance to contemporary digital contexts, it may exclude earlier foundational research on mothers’ literacy or information practices, limiting historical perspective.

### Ethics and dissemination

This scoping review does not require ethical approval, because it solely relies on existing published literature and will not collect any human participants’ data. This approach is consistent with the principles of the Declaration of Helsinki [31], given that no privacy concerns are involved in the use of publicly available data.

The final findings of this review will be presented in peer-reviewed journals and presented at relevant academic conferences. The goal is to clarify the current state of research on mothers’ MIL, identify knowledge gaps, and provide a foundation for future research directions and methodological developments.

## Supporting information

S1 Appendix Search strategy for each database. (PDF)

S2 Appendix PRISMA-P checklist. (PDF)

## References

[1] Dadakhonov AO. Analysis of media and information literacy definitions: A qualitative approach. Stud Media Commun. 2024;12(2):116.

[2] Limilia P, Bono B. Information access skills in mothers as containment of internet negative impact. J Messanger. 2018;10(1):72–82.

[3] Adjin-Tettey T. Combating fake news, disinformation, and misinformation: Experimental evidence for media literacy education. Cogent Arts Human. 2022;9(1):2037229.

[4] Al Zou’bi RM. The impact of media and information literacy on students’ acquisition of the skills needed to detect fake news. J Media Literacy Educ. 2022;14(2):58–71.

[5] Singh N, Banga G. Media and information literacy for developing resistance to ‘infodemic’: Lessons to be learnt from the binge of misinformation during COVID-19 pandemic. Media Cult Soc. 2022;44(1):161–71.

[6] Rahayu NW, Haningsih S. Digital parenting competence of mother as informal educator is not inline with internet access. Int J Child-Comput Interact. 2021;29:100291.

[7] Research E. Moms and media; 2023. https://www.edisonresearch.com/wp-content/uploads/2023/05/Moms-and-Media-2023.pdf

[8] Haywood A, Sembiante S. Media literacy education for parents: A systematic literature review. J Media Literacy Educ. 2023;15(3):79–92.

[9] Angeliqa F, Sarwono BK. Family disposition related to media literacy for advertisements of children’s snacks in the media. J Komun: Malays J Commun. 2019;35(4):258–71.

[10] Lorenzo-Blanco EI, Unger JB, Thrasher JF. E-cigarette use susceptibility among youth in Mexico: The roles of remote acculturation, parenting behaviors, and internet use frequency. Addict Behav. 2021;113:106688. doi: 10.1016/j.addbeh.2020.106688 33053455 PMC7814416

[11] Daneels R, Vanwynsberghe H. Mediating social media use: Connecting parents mediation strategies and social media literacy. Cyberpsychol: J Psychosoc Res Cyberpsychsp-Brno. 2017;11(3):5.

[12] Livingstone S, Blum-Ross A, Pavlick J, Ólafsson K. In the digital home, how do parents support their children and who supports them?; 2018. https://eprints.lse.ac.uk/87952/1/Livingstone_Parenting

[13] Lee SB, Kim S. The structural relationship between mothers’ smart media literacy, smart media mediation, young children’s overdependence on smart devices, and executive function. Korean J Child Stud. 2022;43(3):303–18.

[14] Liana C, Soemardjo HA. Media literacy in the family (descriptive study of parents’ actions of SDIT Alfauzien Depok students in assisting the use of media in children). COMMUSTY J Commun Stud Soc. 2022;1(1):27–37.

[15] Wang Y, Zhao Y, Lu J, Gao Y. Young children’s digital literacy practices with caregivers in the home environment: Voices of Chinese parents and grandparents. Sustainability. 2024;16(8):3300.

[16] Kinahan AM. Cultivating the taste of the nation: The National Council of Women of Canada and the campaign against “pernicious” literature at the turn of the twentieth century. Can J Commun. 2007;32(2):161–79.

[17] Potter WJ. Media literacy. Eighth Edi ed. 2016.

[18] Potter WJ. Children as a special audience. In: Byrnie MJM, Piccininni G, editors. Media literacy. 8th ed. Los Angeles, USA: Sage Publications; 2016. p. 177.

[19] Onishi R, Tone H, Maruyama F, Kubota M, Chino N. Identifying and comparing types of social comparisons on social networking sites among mothers with infants: Differences in maternal loneliness by types. Soc Psychiatry Psychiatr Epidemiol. 2025;60(4):905–15. doi: 10.1007/s00127-024-02677-3 38724742

[20] Khamis S, Agboada DJ. Maternal health information disparities amid Covid-19: Comparing urban and rural expectant mothers in Ghana. Media Commun. 2023;11(1):173–83.

[21] MacPherson AR, Reid M, Dautovich N. Examining the postpartum period through social media: A content and thematic analysis of # postpartum Instagram posts. Psychol Popular Media. 2023;12(4):424.

[22] Jia Y. A study of contemporary Chinese parenting anxiety under the perspective of new media [in Chinese]. Hebei University; 2019.

[23] Chaomeng Z. A case study of new media use and media literacy among middle-aged rural women: A survey report from J village, Henan Province [in Chinese]. Guang Xi University; 2022.

[24] Peters MDJ, Godfrey C, McInerney P. Chapter 10: Scoping reviews; 2024. Available from: https://jbi-global-wiki.refined.site/space/MANUAL/355862497/10.+Scoping+reviews

[25] UNESCO. Global media and information literacy assessment framework: Country readiness and competencies; 2013. https://unesdoc.unesco.org/ark:/48223/pf0000224655

[26] Tricco AC, Lillie E, Zarin W, O’Brien KK, Colquhoun H, Levac D, et al. PRISMA extension for scoping reviews (PRISMA-ScR): Checklist and explanation. Ann Intern Med. 2018;169(7):467–73. doi: 10.7326/M18-0850 30178033

[27] Shamseer L, Moher D, Clarke M, Ghersi D, Liberati A, Petticrew M, et al. Preferred reporting items for systematic review and meta-analysis protocols (PRISMA-P) 2015: Elaboration and explanation. BMJ. 2015;349. 10.1136/bmj.g7647 25555855

[28] Munn Z, Aromataris E, Tufanaru C, Stern C, Porritt K, Farrow J, et al. The development of software to support multiple systematic review types: The Joanna Briggs Institute System for the Unified Management, Assessment and Review of Information (JBI SUMARI). Int J Evid Based Healthc. 2019;17(1):36–43. doi: 10.1097/XEB.0000000000000152 30239357

[29] Page MJ, McKenzie JE, Bossuyt PM, Boutron I, Hoffmann TC, Mulrow CD, et al. The PRISMA 2020 statement: An updated guideline for reporting systematic reviews. BMJ. 2021;372. 10.1136/bmj.n71 PMC8005924 33782057

[30] Peters MDJ, Marnie C, Tricco AC, Pollock D, Munn Z, Alexander L, et al. Updated methodological guidance for the conduct of scoping reviews. JBI Evid Implement. 2021;19(1):3–10. doi: 10.1097/XEB.0000000000000277 33570328

[31] World Medical Association. World Medical Association Declaration of Helsinki: Ethical principles for medical research involving human subjects. JAMA. 2013;310(20):2191–4. doi: 10.1001/jama.2013.281053 24141714

