# Supplementary material for: Media and information literacy among mothers in the 21st century: A scoping review protocol
Source: PLoS One. 2025 Oct 9;20(10):e0333890. doi: 10.1371/journal.pone.0333890 (PMC12510655; doi:10.1371/journal.pone.0333890)
Supplement: S1 Appendix — (PDF) [file pone.0333890.s001.pdf]

SCOPUS<2004 to 4, 2025>

Search conducted on April 16, 2025

Search	Query	Records retrieved
#1	TITLE-ABS-KEY=("media and information literac*" OR "media literac*" OR "information literac*" OR "digital literac*" OR "news literac*" OR "social media literac*" OR "computer literac*" OR "technological literac*" OR "visual literac*" OR "ICT literac*" OR "information and communication technology literac*")	34152
#2	TITLE-ABS-KEY=(mother$ OR "female parent$" OR mommy OR mummy OR mom OR mum)	512023
#3	#1 AND #2	163

wos<2004 to 4, 2025>

Search conducted on April 16, 2025

Search	Query	Records retrieved
#1	Topic=("media and information literac*" OR "media literac*" OR "information literac*" OR "digital literac*" OR "news literac*" OR "social media literac*" OR "computer literac*" OR "technological literac*" OR "visual literac*" OR "ICT literac*" OR "information and communication technology literac*")	24390
#2	Topic=(mother$ OR "female parent$" OR mommy OR mummy OR mom OR mum)	559731
#3	#1 AND #2	146

EBSCO<2004 to 4, 2025>

Search conducted on April 16, 2025

Search	Query	Records retrieved
#1	SU=("media and information literac*" OR "media literac*" OR "information literac*" OR "digital literac*" OR "news literac*" OR "social media literac*" OR "computer literac*" OR "technological literac*" OR "visual literac*" OR "ICT literac*" OR "information and communication technology literac*")	93853
#2	SU=(mother$ OR "female parent$" OR mommy OR mummy OR mom OR mum)	387291
#3	#1 AND #2	58

Taylor&Francis <2004 to 4, 2025>

Search conducted on April 16, 2025

Search	Query	Records retrieved
#1	Anywhere=("media and information literac*" OR "media literac*" OR "information literac*") OR "digital literac*" OR "news literac*" OR "social media literac*" OR "computer literac*" OR "technological literac*" OR "visual literac*" OR "ICT literac*" OR "information and communication technology literac*")	4
#2	Anywhere=(mother$ OR "female parent$" OR mommy OR mummy OR mom OR mum)	1074544
#3	#1 AND #2	1
